# Blocking TGF-β Signaling Pathway Preserves Mitochondrial Proteostasis and Reduces Early Activation of PDGFRβ+ Pericytes in Aristolochic Acid Induced Acute Kidney Injury in Wistar Male Rats

**DOI:** 10.1371/journal.pone.0157288

**Published:** 2016-07-05

**Authors:** Agnieszka A. Pozdzik, Laetitia Giordano, Gang Li, Marie-Hélène Antoine, Nathalie Quellard, Julie Godet, Eric De Prez, Cécile Husson, Anne-Emilie Declèves, Volker M. Arlt, Jean-Michel Goujon, Isabelle Brochériou-Spelle, Steven R. Ledbetter, Nathalie Caron, Joëlle L. Nortier

**Affiliations:** 1 Laboratory of Experimental Nephrology, Department of Biochemistry, Faculty of Medicine, ULB, Brussels, Belgium; 2 Nephrology Department, Erasme Hospital, ULB, Brussels, Belgium; 3 Laboratory of General Physiology, URPHYM, University of Namur, Namur, Belgium; 4 CardioMetabolic and Renal Research, Cell Biology, Genzyme Corporation, Framingham, Massachusetts, United States of America; 5 Pathology and Electron Microscopy, CHU La Miletrie, Poitiers, France; 6 INSERM U 1082, Poitiers, France; 7 Analytical and Environmental Sciences Division, MRC-PHE Centre for Environment and Health, King’s College London, London, United Kingdom; 8 Pathology Department, Tenon Hospital, Paris, France; Biogen Idec, UNITED STATES

## Abstract

**Background:**

The platelet-derived growth factor receptor β (PDGFRβ)+ perivascular cell activation becomes increasingly recognized as a main source of scar-associated kidney myofibroblasts and recently emerged as a new cellular therapeutic target.

**Aims:**

In this regard, we first confirmed the presence of PDGFRβ+ perivascular cells in a human case of end-stage aristolochic acid nephropathy (AAN) and thereafter we focused on the early fibrosis events of transforming growth factor β (TGFβ) inhibition in a rat model of AAN.

**Materials and Methods:**

Neutralizing anti-TGFβ antibody (1D11) and its control isotype (13C4) were administered (5 mg/kg, i.p.) at Days -1, 0, 2 and 4; AA (15 mg/kg, sc) was injected daily.

**Results:**

At Day 5, 1D11 significantly suppressed p-Smad2/3 signaling pathway improving renal function impairment, reduced the score of acute tubular necrosis, peritubular capillaritis, interstitial inflammation and neoangiogenesis. 1D11 markedly decreased interstitial edema, disruption of tubular basement membrane loss of brush border, cytoplasmic edema and organelle ultrastructure alterations (mitochondrial disruption and endoplasmic reticulum edema) in proximal tubular epithelial cells. Moreover, 1D11 significantly inhibited p-PERK activation and attenuated dysregulation of unfolded protein response (UPR) pathways, endoplasmic reticulum and mitochondrial proteostasis *in vivo* and *in vitro*.

**Conclusions:**

The early inhibition of p-Smad2/3 signaling pathway improved acute renal function impairment, partially prevented epithelial-endothelial axis activation by maintaining PTEC proteostasis and reduced early PDGFRβ+ pericytes-derived myofibroblasts accumulation.

## Introduction

Tubulointerstitial fibrosis is a multistage process arising form different causes closely related to the progression of all chronic kidney diseases (CKD). [1, 2] Both tubular epithelial and endothelial cells (EC) by forming epithelial-endothelial axis together with dendritic and interstitial mesenchymal cells are involved in kidney scaring. [3–5] This axis serves as a resident cellular unit sensing and mediating the renal insult regardless of its origin (ischemia, toxicity, inflammation). Moreover, endoplasmic reticulum (ER) stress and activation of mitochondrial unfolded protein response (UPR) pathway transmit danger signals out of these organelles to the nucleus of tubular as well as EC [6] and accelerate fibrotic remodeling. [7, 8]

Contribution of tubular and EC throughout their transdifferentiation into the mesenchymal cells (EMT and endoMT, respectively) in renal myofibroblasts generation remains controversial. [5, 9] Recently, the pericytes and resident fibroblasts (interstitial mesenchymal cells attached to the peritubular capillaries and to the tubules, respectively) became increasingly recognized as the main source of scar-associated kidney myofibroblasts. [10–14] They emerged as a new cellular therapeutic target for chronic kidney disease (CKD). [15] Indeed, deregulation of pericytes homeostasis alters epithelial-endothelial axis, leads to the modification of peritubular capillaries network and alters cells regeneration, mainly in proximal tubular epithelial cells (PTEC) because of their high cellular metabolism and physiologically low oxygen delivery. [1, 2, 16] Furthermore, PTEC cycle arrest, microvasculature injury and growth factors have been reported most likely as triggers for pericytes and resident fibroblasts differentiation into myofibroblasts. [17–20]

Transforming growth factor beta (TGFβ) is a pivotal profibrotic mediator responsible of renal scaring. [21] Enhanced expression of platelet-derived growth factor (PDGF) and its receptor β characterizes all experimental and human fibrotic kidney diseases. [22] In obstructive and ischemic kidney fibrosis, TGFβ induces profibrotic signaling in tubular epithelial cells, stimulates PDGFRβ+ pericytes activation, proliferation, and transition into the myofibroblasts. [23, 24] Interventions to inhibit TGFβ signaling pathways have been successful to reduce renal fibrosis and are well tolerated in animal models. [25–33] However, the role of TGFβ/Smad inhibition in the setting of PDGFRβ+ perivascular cells in AKI remains controversial and depends on the investigated model. [31, 34]

Aristolochic acid nephropathy (AAN) is characterized by marked tubular atrophy and typical paucicellular renal fibrosis. [35–37] In our previous experimental studies in a rat model of AAN, we identified two distinct phases. An early acute phase from day 0 to day 5, which is characterized by acute PTEC necrosis accompanied by an increased expression of TGFβ, activation of p-Smad2/3 initially mainly in the areas of external part of medullary rays, and a chronic phase from day 6, which is mostly represented by interstitial cells infiltration leading progressively to marked tubular atrophy and renal fibrosis at day 35, as attested by alpha smooth muscle actin (αSMA) positive myofibroblasts and collagens deposition in the vicinity of damaged proximal tubules. [38] Increased expression of TGFβ and p-Smad2/3 has initially been observed in PTEC and then in the interstitial cells, mainly in the areas of external part of medullary rays. [38, 39] We earlier hypothesized that PTEC are responsible for sensing and activating transition of resident fibroblasts into myofibroblasts in AA-induced fibrosis. [37] As we detected signs of peritubular neoangiogenesis during the acute phase, mainly at day 5 (unpublished data), actually we made the hypothesis that an early activation of interstitial perivascular cells could represent an important step in the onset of AA induced fibrosis.

Here, we firstly confirmed the involvement of PDGFRβ+ perivascular cells in AA-induced renal fibrosis in humans using kidney tissue specimen from a case of end-stage AAN. Thereafter, we examined the effects of TGFβ signaling pathway inhibition on acute TI injury and interstitial perivascular cell activation induced by AA *in vivo*. To this end, male Wistar rats were preconditioned with mouse anti-TGFβ antibody (Ab) (1D11) [40], which neutralizes all three mammalian TGFβ isoforms. In these settings, we assessed the impact of TGFβ inhibition on outcome of renal function, acute ultrastructure alterations of epithelial-endothelial axis and interstitial perivascular cells activation induced by AA.

## Materials and Methods

All procedures were approved by the local Ethic Committee for Animal Care and were conducted according to the National Guidelines for the Care and Use of Laboratory Animals. This study was carried out in strict accordance with the recommendation in the Guide for the Care and Use of Laboratory Animals of the National Institutes of Health. The protocol has been approved by the Committee on the Ethics of Animal Experiments of the Université Libre de Bruxelles (1230326).

### Experimental protocols

All experimental procedures were performed in 5 weeks-old male Wistar rats (Elevage Janvier, Le Genest Saint-Isle, France). [38, 39, 41] Animals were housed in a monitored temperature, humidity and light environment in the animal care facility of Brussels University (Brussels, Belgium). They had free access to water and food. After one week of acclimatization, weight-matched rats were randomly assigned to study groups (n = 6 per each group). We have undertaken all efforts to minimize potential pain and distress. No animals became severely ill.

Mouse pan-anti-TGFβ Ab (1D11) and its isotype control Ab (13C4) were obtained from Genzyme Corporation (New York, USA). Accordingly to the expert opinion and after preliminary study we used the dose of 5 mg/kg of 1D11 and 13C4. The AA mixture (15 mg/kg) was dissolved in PEG; PEG (equivalent volume of AA injection) was injected sc once daily while the two tested antibodies were injected i.p. at Days -1, 0, 2 and 4. We studied 4 groups of weight-matched rats (n = 6) randomly assigned: (1) control group (PEG + neutralizing anti-TGFΒ Ab), [42] AA group (AA+PEG), (3) AA + neutralizing anti-TGFβ group (AA + anti-TGFβ Ab) and (4) AA + control Ab (AA + isotype control Ab).

The animals were put in metabolic cages for 24 h urine collections the day before the experiment. Urine samples were centrifuged at 1600 g at ambient temperature for 15 min and stored at -20°C for further measurement.

Blood samples were obtained by cardiac puncture and were centrifuged at 1600 g at 4°C for 15 min. The samples of urine and plasma were stored at -20°C until further analysis. [38, 39, 41] On Day 5, surgery was performed. Animals were anesthesized with an injection of ketamine HCl (44 mg/kg, i.p.) (Merial, Brussels, Belgium) and xylazine 2% (6 mg/kg, i.p.) (Bayer, Brussels, Belgium) after 5 days of protocol. Both kidneys were harvested. One half of the left kidney was fixed in Dubosq-Brazil solution and the other half in a 4% paraformaldehyde buffered solution (pH 7.4). Both halves were embedded in paraffin for further evaluation. We took one tissue sample of cortex, OSOM and ISOM form the right kidney as described previously. [43] Specimens were immediately frozen (‒80°C) for protein extraction.

### Western blotting

Kidney lysates were electrophoresed on SDS-PAGE gels (Invitrogen Inc., Grand Island, NY USA) and transferred to nitrocellulose membranes. These membranes were blocked in TBST containing 5% non-fat milk for 1 h (room temperature). Immunoblotting was performed with primary antibodies: anti-p-Smad2, anti-p-Smad3, anti-VEGFR2, and anti-PDGFR beta and anti-GAPDH, anti-SDHA, anti-ATPB, anti-p-PERK, anti-p-eIF2α, anti-TeIF2α (Cell Signaling Technology Inc., Danvers, MA, USA), anti-TGFRII (Santa-Cruz Biotechnology, Inc, Dallas, TX, USA), at 4°C overnight. Next, horse peroxidase-labelled appropriate secondary antibodies were added. The plotted proteins were visualized by ECL detection system (Thermo Fisher Scientific, Rockford, IL USA). Densitometric analysis was performed using ImageJ version 1.440.

### Biochemical evaluation of renal function

Plasma and urinary creatinine levels, urinary activity of *N*-acetyl-β-glucosaminidase (NAG) were measured as detailed previously. [38, 39, 41]

### Kidney histological analysis

Kidney coronal sections (5 μm) were stained with hematoxylin/eosin and periodic acid-Schiff for quantification of the TI injury as reported previously. [44] Three investigators (L.G., A.P. and I.B.) blind to rat’s group origin, performed analyses independently.

### Immunohistochemistry

All immunostainings of following primary antibodies: monoclonal anti-αSMA, polyclonal goat anti**-**vimentin and polyclonal rabbit anti-vWF (DakoCytomation, Heverlee, Belgium), monoclonal mouse anti-neutral endopeptidase, monoclonal anti-fibronectin (Lab Vision, Fremont, CA, USA), polyclonal rabbit anti-caspase 3 and rabbit monoclonal anti-PDGF receptor β (Cell Signaling Technology, Leiden, The Netherlands) were performed as extensively detailed previously. [37–39, 41]

Two independent investigators (L.G. and A.P.) blind to the group origin of the rats performed all quantifications. Quantification of vWF+ peritubular capillaries were evaluated at x40 magnification in 30 fields (2.52 mm² of kidney tissue) and expressed as the number of vWF+ peritubular capillaries per field. Quantification of αSMA and vimentin immunostainings was performed using computer-assisted morphometric analysis (ImageJ-National Institute of Health, city, USA).

### Ultrastructural analysis

Analysis of ultrastructure using transmission electron microscopy was performed in the same period as previously described. [45]

### Human proximal epithelial cell (HK-2) and human umbilical vein endothelial cell (HUVEC) culture

Human umbilical vein endothelial cells (HUVECs) and human proximal epithelial cells (HK-2) (Lonza, Verviers, Belgium) were maintained according to the manufacturer’s instructions. When almost reaching confluence, cells were trypsinized and transferred to 4-well plates (5×10^4^ cells/well).

HUVECs or HK-2 (4×10^4^ cells/well) were seeded in 96-well plates. After incubation for 24 h, AA or TGFβ in presence or absence of anti- TGFβ were added to each well and cells were cultured for 6 or 24 h. To assess cell viability the MTT assay (The CellTiter 96®, Promega USA) was used. Cells were switched to MTT solution (5 mg/mL) for 4 h. Finally, 100 μL of DMSO was added to each well and absorbance of the solution was measured at 490 nm using iEMS Reader MF spectrophotometer (Thermo Labsystem, Finland).

### Statistical analysis

All data were compared between all groups by parametric one-way ANOVA test with Posthoc Holm-Sidak analyses. Different significance levels (*** *P* < .001, ** *P* < .01, * *P* < .05) were applied.

## Results

### Interstitial perivascular cells expressing PDGFRβ accumulated in human end-stage AAN

**Fig 1:**
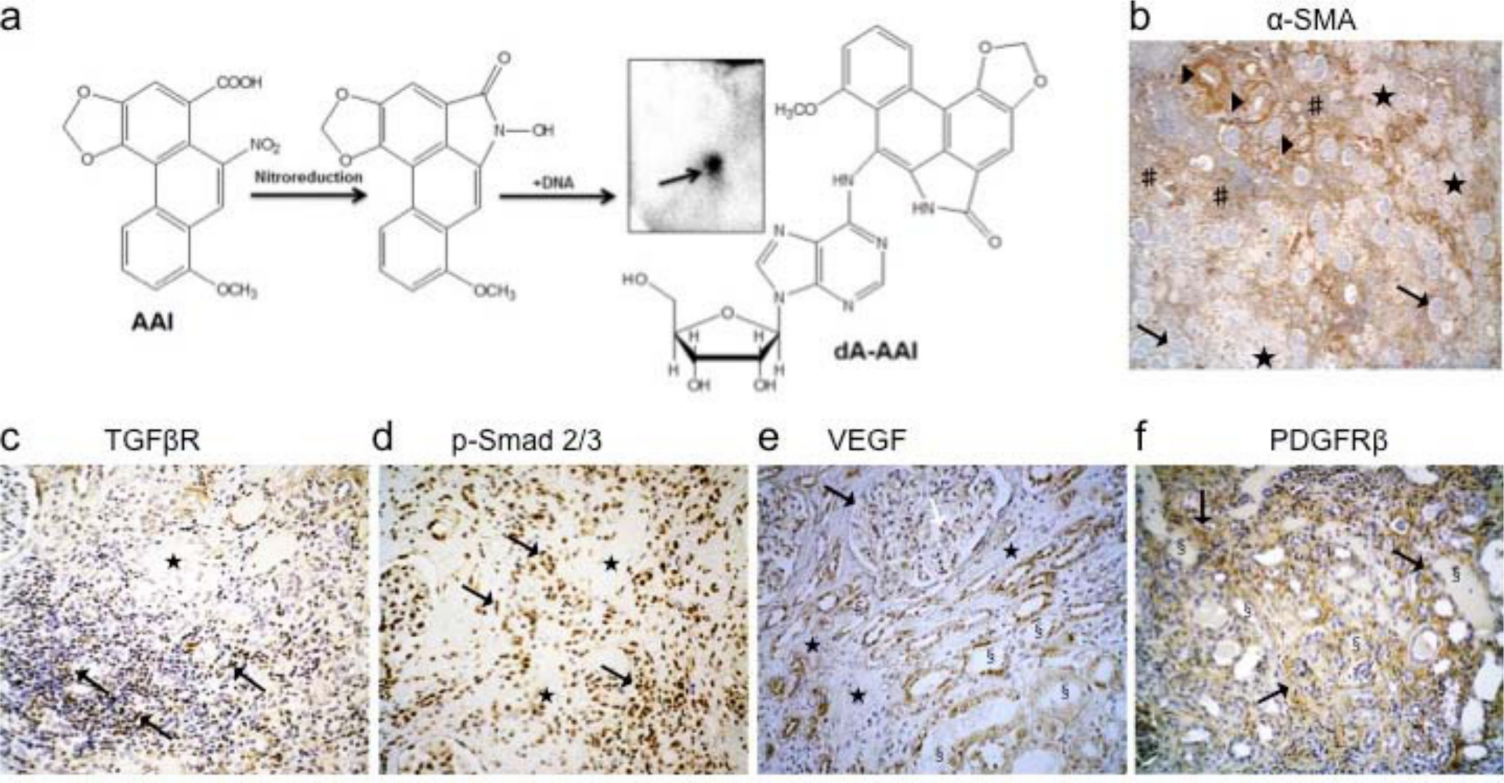
Representative histological findings in a human case of end-stage aristolochic acid nephropathy (AAN). A 59-years old woman with end-stage AAN was admitted in April 2005 to the nephrology department for the prophylactic surgical removal of native kidneys and ureters considering the high risk of urothelial carcinoma linked to AA exposure. She was on the waiting list for kidney transplantation. Renal replacement therapy consisted of ambulatory hemodialysis (4hx3 times per week). Both kidneys were atrophic (8.5 cm measurement by ultrasonography). Ureterobinephrectomy was performed by caelioscopy. Macroscopically, kidneys showed a typical reduction of cortical area and of weight (right and left kidney: 155 g and 150 g, respectively). (**a**) Using the ^32^P-poslabelling assay specific 7-(deoxyadenosin-*N*^6^-yl)-aristolactam I (dA-AAI) adducts (0.36 ± 0.13 adducts per 10 ^7^ normal nucleotides) were detected in the renal cortex homogenate which confirmed previous exposure to AA; the insert shows the dA-AAI adduct on the thin-layer chromatography plate after autoradiography. (**b**) Tissue expression of alpha-smooth muscle actine (α-SMA) was observed within vessels walls (▶) and by several interstitial cells (#) predominantly outside of fibrotic areas (★). Glomeruli were well-preserved glomeruli (→). (**c**) Several interstitial cells expressed receptor of transforming growth factor beta (TGFβ) (CD105-endoglin: →) and (**d**) phosphorylated Smad2/3 (→) around fibrotic areas (★). (**e**) Besides podocytes marked expression of vascular endothelial growth factor (VEGF) (→) was found in several atrophic tubular cells (§) typically around fibrotic areas (★). (**f**) Cells expressing platelet-derived growth factor receptor beta (PDGFRβ) (→) massively accumulated in the interstitial areas around atrophic tubules (§). Immunohistochemical analysis of human formalin-fixed paraffin-embedded kidney tissue sections (immunoperoxidase technic counterstained with hematoxylin) using anti-human antibodies recognizing: (**b**) alpha-SMA (mouse monoclonal, Dako, Heverlee, Beglium), (**c**) TGFβR (CD105, endoglin) (mouse monoclonal, Thermo Scientific, Fremont CA, USA), (**d**) phosphorylated Smad2/3 (rabbit polyclonal affinity purified, Santa-Cruz Biotechnology, Inc, Dallas, TX, USA), (**e**) VEGF (rabbit polyclonal, Thermo Scientific, Fremont CA, USA), (**f**) PDGFRβ (rabbit monoclonal, Cell Signaling Technology, Leiden, The Netherlands). Original magnification: **b**: x40; **c**, **d** and **f**: x100; **e**: x200.

**Fig 2:**
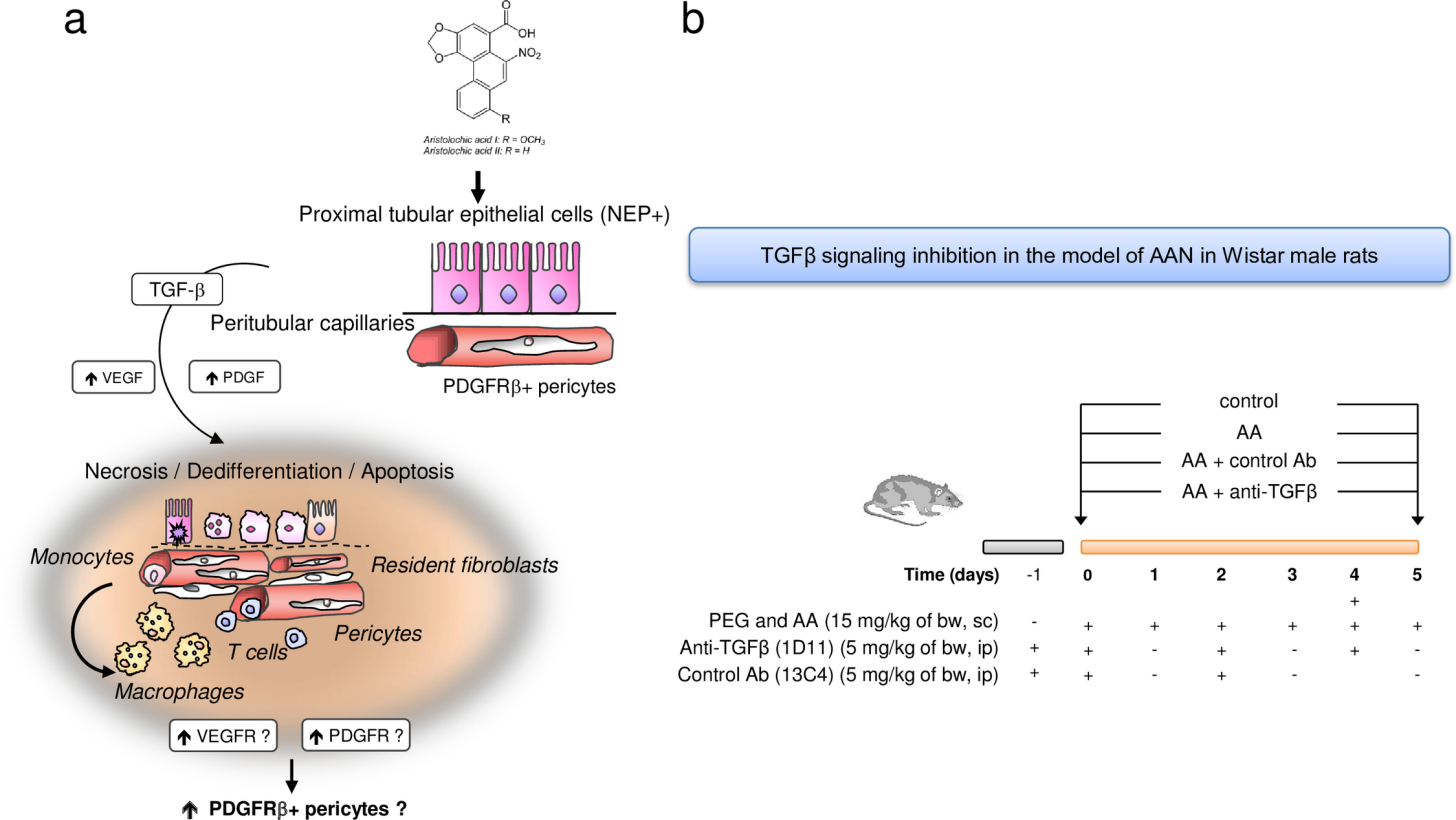
Schema showing the proposed hypothesis of tubular and vascular injury and perivascular cells involvement in the pathogenesis of aristolochic acid (AA)-related kidney fibrosis. (**a**) Sustained exposure to AA is harmful for proximal tubular epithelial cells (PTEC) leading to incomplete proliferation of PTEC resulting in apoptosis inhibition. These surviving cells secrete transforming growth factor beta (TGFβ) among several other pro-inflammatory and pro-fibrotic paracrine factors. In this microenvironment, kidney perivascular cells (resident fibroblasts and pericytes) expressing platelet-derived growth factor receptor beta (PDGFRβ) are activated into interstitial myofibroblasts. Peritubular capillary damage can occur also after AA intoxication or secondary to dysregulation of pericytes homeostasis. (**b**) Design of study using anti-TGFβ antibody (1D11) evaluating the effects of TGFβ signaling pathway inhibition on tubular, endothelial and perivascular cells injuries in acute phase of AAN in Wistar rats.

Using ^32^P-postlabelling AA-specific DNA adducts (i.e. 7-(deoxyadenosin-*N*^6^-yl)-aristolactam I; dA-AAI) were detectable in the kidney cortex DNA of this patient (Fig 1A) confirming the exposure to AA accordingly to previous reports. [46, 47] Several α-SMA, TGFβ receptors and p-Smad2/3 positive interstitial cells mainly bordered the fibrotic scars and reflected activation of myofibroblasts and p-Smad2/3 signaling pathway, respectively (Fig 1B–1D). Numerous atrophic cells from the remaining tubules outside fibrotic scars expressed vascular endothelial growth factor (VEGF) (Fig 1E). In the adjacent interstitium, an increase in PDGFRβ staining suggested the accumulation of perivascular cells (resident fibroblasts and pericytes) (Fig 1F). Considering these histological findings, we studied the hypothesis of the early involvement of PTEC as well as of peritubular capillaries injury triggering interstitial perivascular cells through TGFβ signaling pathway in AA-related fibrogenesis (Fig 2A and 2B).

### Anti-TGFβ Ab inhibited the activation of AA-induced p-Smad2/3 signaling pathway

**Fig 3:**
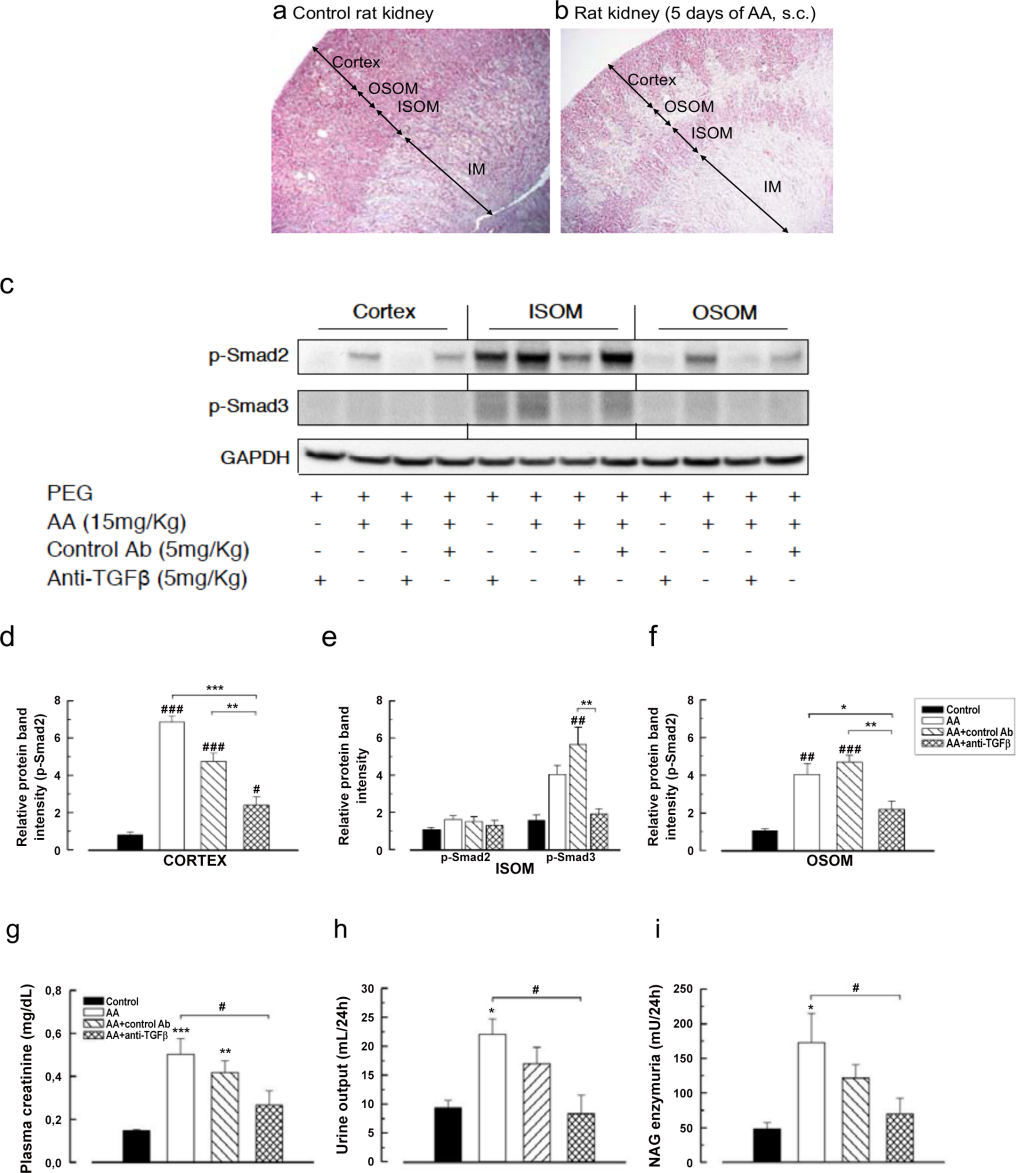
Anti-transforming growth factor beta (TGFβ) Ab suppressed p-Smad2/3 signaling in the kidney induced by aristolochic acid (AA) and attenuated acute kidney injury. Representative photomicrographs of longitudinal kidney section (**a**) in rat control and (**b**) in rat receiving aristolochic acid (AA) during 5 days. Arrows depict areas of cortex, of outer stripe of outer medulla (OSOM), of inner stripe of outer medulla (ISOM) and of inner medulla (IM). Please note that AA induced severe acute tubulointerstitial injury in the medullary rays. (**c**) Tissue lysates from cortex, OSOM, and ISOM were immunoblotted for p-Smad2, p-Smad3, and glyceraldehyd 3-phosphate dehydrogenase (GAPDH) expression. Bands intensities of p-Smad2 protein in studied groups (n = 3 for controls; n = 4 for AA group; and n = 5 for AA+control isotype Ab and AA+anti-TGFβ groups) were quantified by densitometry. (**d-f**) The control group displayed a low basal level of p-Smad2/3 activation, and anti-TGF Ab had a protective effect. Results are presented as means ± SEM. One way ANOVA, *** *P* < .001, ** *P* < .01, * *P* < .05 comparison of each group versus control group; followed by Holm Sidak test, between groups ### *P* < .001, ## *P* < .01, # *P* < .05. Protective effects on AA-induced functional parameters: (**g**) increase in plasma creatinine level and (**h**) polyuria and (**i**) proximal tubular cells structural abnormalities reflected by *N*-acetyl-β-glucosaminidase (NAG) enzymuria. Data are shown as mean ± SEM (n = 6, except for AA+control Ab n = 5). *** *P* < .001, ** *P* < .01, * *P* < .05 comparison of each group versus control group; followed by Holm Sidak test, # *P* < .05 comparison between all groups.

In experimental AAN, we reported earlier collagens type III and I patchy deposition in the external parts of medullary rays in the chronic phase. [39] In the present study, the topography of acute tubular necrosis correlated with the fibrotic areas (Fig 3A and 3B). Therefore, we assessed p-Smad2 and p-Smad3 in kidney lysates from the cortex, the outer and inner stripe of outer medulla (OSOM and ISOM respectively). AA activated p-Smad2 and p-Smad3 in all of them (Fig 3C–3F). Anti-TGFβ Ab (1D11), but not control isotype Ab (13C4), significantly suppressed p-Smad2 expression in the cortex and OSOM (2.40 ± 0.45 *vs* 6.85 ± 0.34, *P* < .001 and 2.19 ± 0.44 *vs* 4.05 ± 0.59, *P* < .05, respectively) and p-Smad3 expression in ISOM (4.04 ± 0.49 *vs* 1.91 ± 0.29, *P* NS). Blocking p-Smad2/3 signaling pathway reduced the plasma creatinine (PCr) increase and polyuria (0.27 ± 0.67 mg/dL *vs* 0.50 ± 0.07 mg/dL, *P* < .05 and 8.3 ± 3.2 mL/24 h *vs* 9.3 ± 1.3 mL/24 h, *P* < .05, respectively), and decreased nearly 2.5-fold NAG enzymuria (*P* < .05) as compared with the AA group (Fig 3G–3I).

### Anti-TGFβ prevented AA-induced acute tubulointerstitial injury

**Fig 4:**
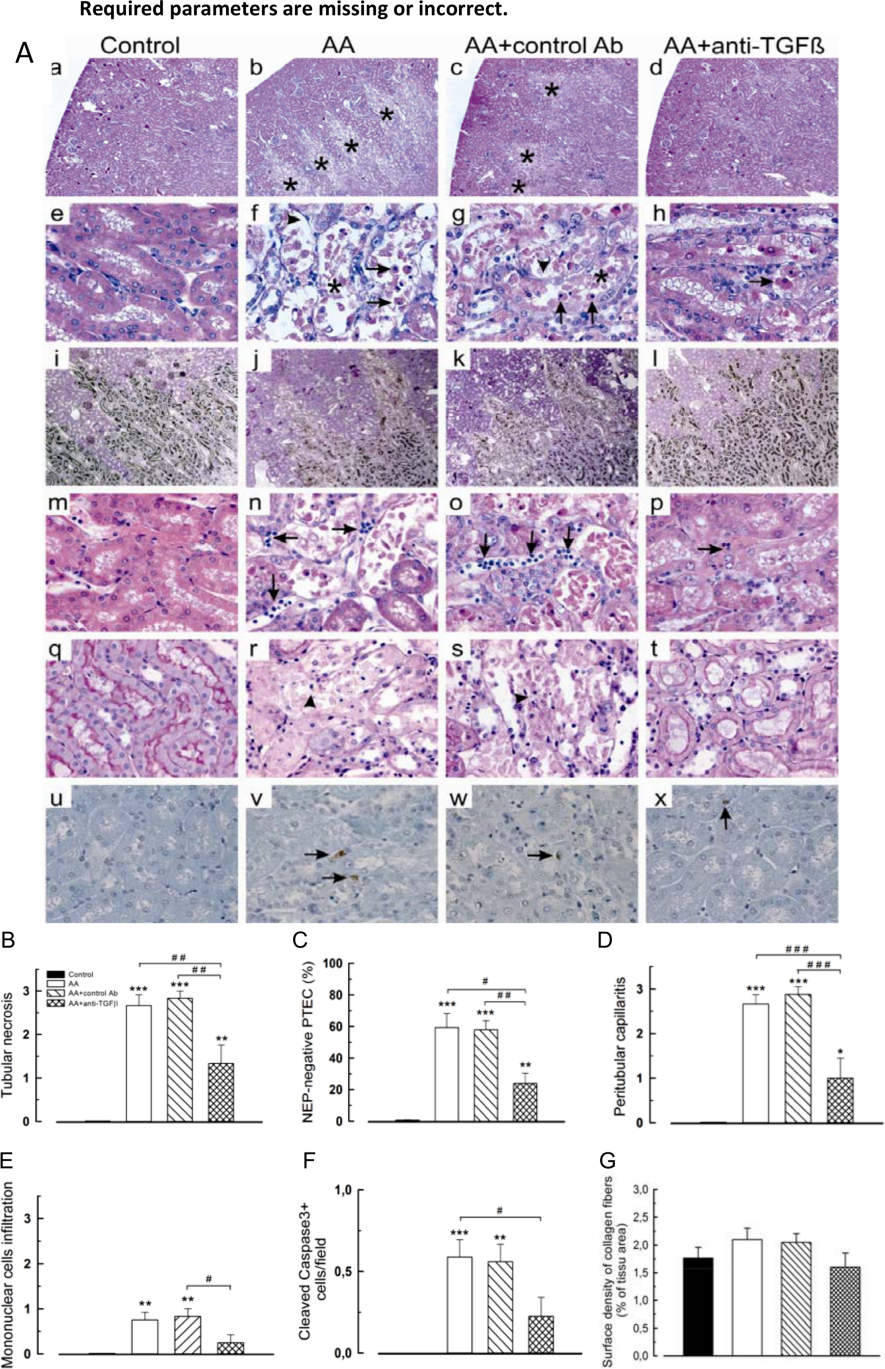
Changes in tubulointerstitial injuries related to aristolochic acid (AA) treatment modulated by anti-transforming growth factor beta (TGFβ) Ab. Anti-TGFβ Ab reduced: (**A**) (**a-d**) areas of proximal tubular epithelial cells (PTEC) necrosis (asterisks), (**e-h**) number of intratubular necrotic cells (arrows) and cellular debris (asterisks) as well as detachment of injured tubular cells (arrowheads). Anti-TGFβ-treated rats exhibited (**i-l**) well-preserved (neutral endopeptidase) NEP expression by PTEC brush border and less (**m-p**) interstitial inflammation. Peritubular capillaritis (arrows) and (**q-t**) disruption of tubular basement membrane (arrowheads) were also attenuated by anti-TGFβ. (**u-x**) Anti-TGFβ reduces cleaved caspase-3 expression. Hematoxylin/eosin (**a-h**, **m-p**), Periodic acid Shiff (**q-t**) stainings, immunohistochemistry of NEP (**i-l**) and of cleaved caspase3 (**u-x**). Original magnifications: **a-d**, x40; **i-l**, x100; **e-h**, **m-t** and **u-x**, x400. NEP: neutral endopeptidase. The scoring system of tubulointerstitial injury was defined as follows: *tubular necrosis*: 0, normal tubules; 1, rare single necrotic tubules; 2, several clusters of necrotic tubules; 3, confluence of necrotic clusters; *interstitial inflammatory infiltrate*: 0, no inflammatory infiltrate or < 10% of inflammatory parenchyma; 1, 10% to 25% of inflammatory parenchyma; 2, 26% to 50% of inflammatory parenchyma; 3, > 50% of inflammatory parenchyma. We scored *peritubular capillaritis* as follows: c0, no inflammation in capillaries or < 10% of cortex capillaries presented inflammatory cells; c1, > 10% of cortex capillaries presented a maximum number of 3 to 4 inflammatory cells in their lumen; c2, > 10% of cortex capillaries presented a maximum number of 5 to 10 inflammatory cells in their lumen; c3, > 10% of cortex capillaries presented more than 10 inflammatory cells in their lumen. Semiquantitative scores of: (**B**) tubular necrosis, (**C**) NEP expression within brush border of PTEC, (**D**) peritubular capillaritis, (**E**) mononuclear cells infiltration, (**F**) PTEC apoptosis and (**G**) collagen fibers deposition. Data are shown as mean ± SEM (n = 6). One way ANOVA, *** *P* < .001, ** *P* < .01, * *P* < .05 comparison of each group versus control group; followed by Holm Sidak test, ### *P* < .001, ## *P* < .01, # *P* < .05 comparison between all groups.

As compared to AA and AA+13C4 groups, 1D11 significantly reduced the extent and severity of PTEC acute necrosis. We observed nearly 2-fold reduction of the semi-quantitative score of acute tubular necrosis (*P* < .01) (Fig 4A, a-h and 4B).

### Polarity of PTEC

The polarity of PTEC was studied through immunostaining of neutral endopeptidase (NEP), a brush-border linked enzyme exclusively expressed by PTEC from S3 segment in rats (OSOM). [48] Anti-TGFβ Ab significantly prevented the loss of NEP expression observed in AA and AA+13C4 groups (Fig 4A, i-l and 4C, *P* < .05).

### Interstitial inflammation, peritubular capillaritis and TBM integrity

As compared to controls, AA induced expansion and edema of the interstitium, severe peritubular capillaritis, mononuclear cell infiltration and tubulitis (Fig 4A, m-o). Anti-TGFβ Ab significantly reduced (3-fold reduction, *P* < .001) peritubular capillaritis as compared to AA and AA+13C4 groups (Fig 4A, n-p and 4D) and reduced mononuclear cell infiltration only as compared to AA+13C4 group (Fig 4A, f-h and 4E).

In contrast to the controls (Fig 4A, q), severe denudation and even rupture of the tubular basement membrane (TBM) occurred in the area of acute tubular necrosis in AA and AA+13C4 groups (Fig 4A, r-s). The integrity of the TBM was preserved in the AA+1D11 group (Fig 4A, t). No difference was found in collagen fiber deposition (Fig 4G), however the power of the performed test was below the desired power indicated that we were less likely to detect a difference when one actually exists.

### Apoptosis of PTEC

Compared to the controls, only some PTEC underwent apoptosis as reflected by weak nuclear staining of activated caspase-3 in AA and AA+13C4 groups (Fig 4A, u-w). Nevertheless, 1D11 (Fig 2D, x) significantly reduced AA-induced apoptosis (0.23 ± 0.12 *vs* 0.59 ± 0.11, *P* < .05, Fig 3F).

### Tubulointerstitial ultrastructure features

**Fig 5:**
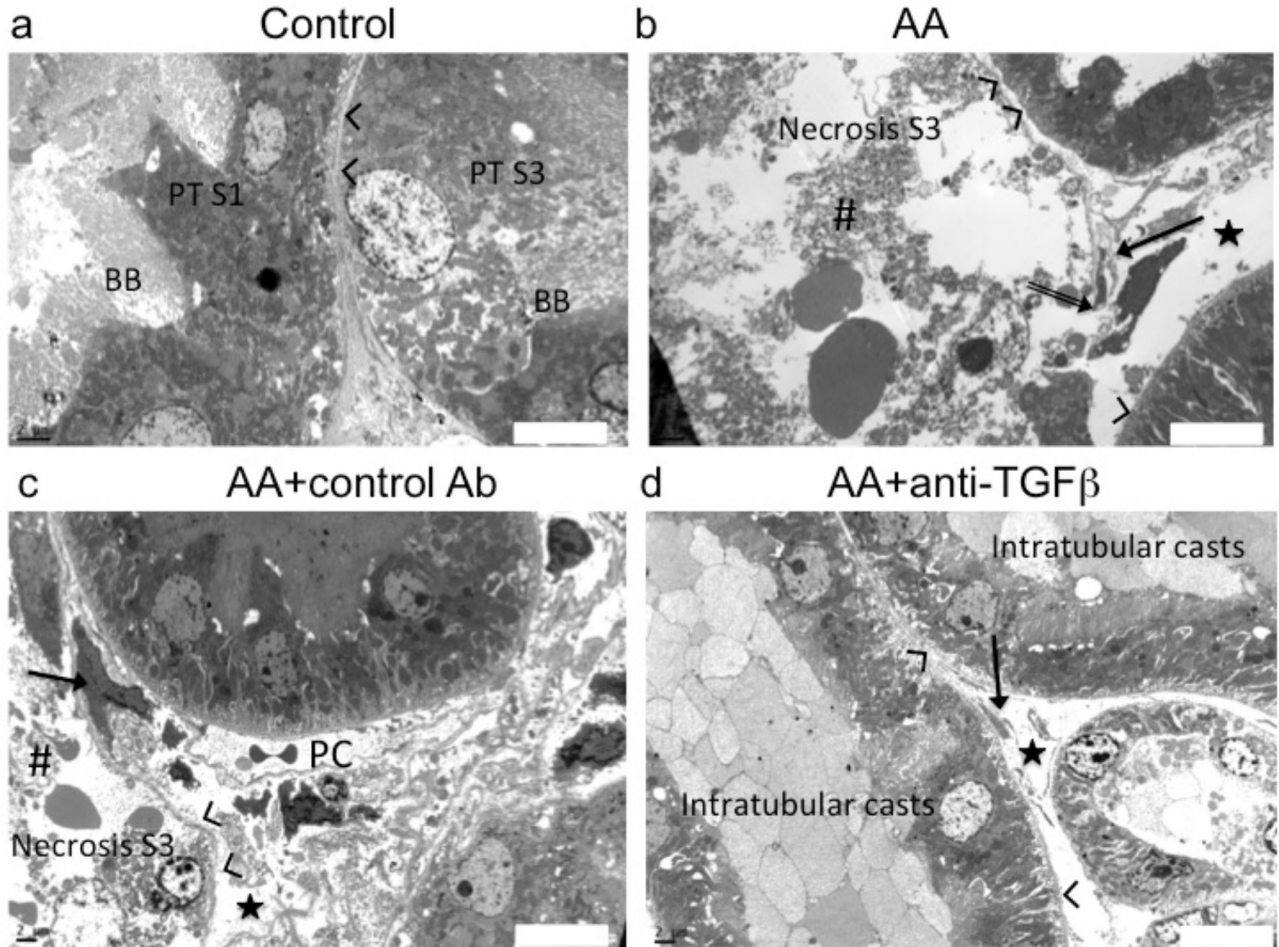
Anti-transforming growth factor beta (TGFβ) Ab attenuated aristolochic acid (AA)-induced ultrastructural alterations within the tubulointerstitial compartment. (**a**) In control rats, S1 and S2 segment tubular cells exhibited prominent features such as the lush carpet of long microvilli, basolateral cell membrane interdigitations and dense network of cytoplasmic microfilaments, numerous long mitochondria and endocytic vacuolae. Tubular cells form S3 segment were cuboidal, had short microvilli and lateral cell membrane digitations restricted to the lower third of the cell height. Some tubular cells contained few edematous mitochondria. (**b** and **c**) In the AA and in the AA+control Ab groups severe acute tubular necrosis was limited to S3 segment. Complete loss of brush border, detachments and lysis of tubular epithelial cells (necrotic cellular debris: #), interstitial exudative edema (stars) accompanying by marked enlargement of interstitium, denudation and rupture (double arrow) of tubular basement membrane (arrow head). Enlarged interstitial peritubular capillaries (PC); resident fibroblast (arrow). (**d**) In the AA+ 1D11 group, less severe injury of proximal tubular epithelial cells and adjacent interstitial edema were observed. Transmission electron microscopy performed on ultrathin (50 nm) kidney tissue sections, original magnification: x3000.

Severe exudative interstitial edema and ATN of S3 segment were confirmed by ultrastructure analyses in AA and AA+13C4 groups (Fig 5A–5C). Preconditioning with 1D11 clearly reduced the extent and severity of S3 segment ultrastructure injuries, preserved the TBM integrity and reduced exudative interstitial edema (Fig 5D).

### Anti-TGFβ Ab modulated kidney accumulation of interstitial myofibroblasts and fibronectin deposition

**Fig 6:**
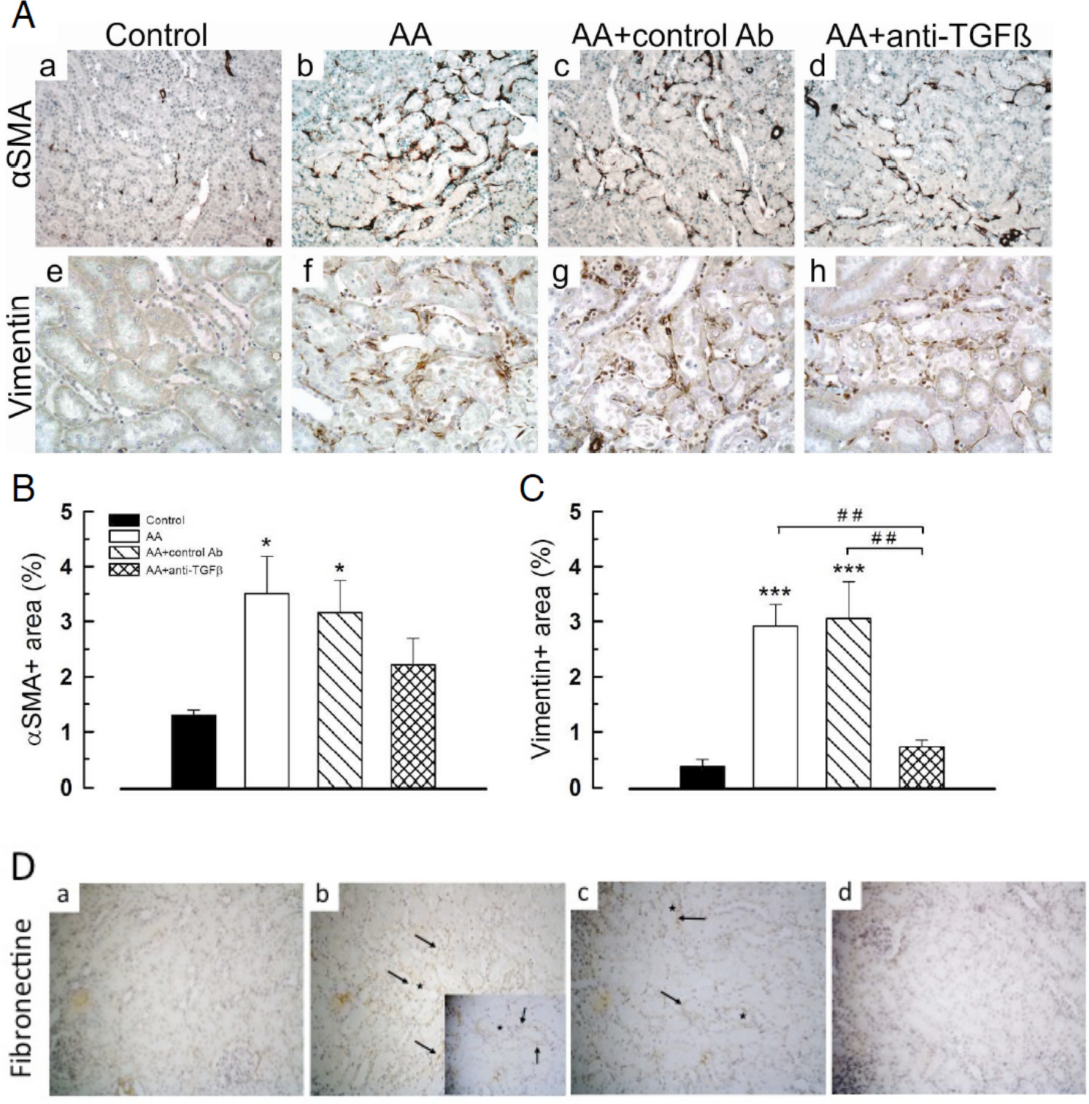
Anti-transforming growth factor beta (TGFβ) Ab modulated accumulation of mesenchymal cells and increase in fibronectin in tubulointerstitial compartment induced by aristolochic acid (AA). (**A**) (**a-d**) As compared to control, enhanced tissue expression of α-SMA and (**e-h**) of vimentin were firmly limited to the external mart of medullary rays. (**B**) and (**C**) Corresponding semi-quantitative scores of α-SMA and vimentin immunostaining, respectively. (**D**) (**a-d**) Tissue expression of fibronectin (arrow) was found mainly around injured tubules and peritubular capillaries. Data are shown as mean ± SEM (n = 6). One way ANOVA, * *P* < .05, ** *P* < .001, *** *P* < .001 comparison of each group versus controls; followed by Holm Sidak test, ## *P* < .01 comparison between all groups (n = 4, 5 or 6). Original magnifications: **Aa-h** and **Da-d** x200 (small picture in B x400).

In comparison with controls, AA considerably increased the interstitial expression of α-SMA (3.50% ± 0.68% *vs* 1.30% ± 0.09%, *P* < .05) (Fig 6A, a-d and 6B) as well as vimentin expression (2.92% ± 0.39% and 3.05% ± 0.67% respectively *vs* 0.37% ± 0.13%, *P* < .001) (Fig 6A, e-g). Anti-TGFβ Ab significantly decreased AA-induced expression of vimentin (0.73% ± 0.12%, 2.92 ± 0.39%, *P* < .01) and of α-SMA, reflecting modulation of interstitial myofibroblasts (Fig 6A–6C). Fibronectin is an extracellular matrix component highly up-regulated by TGFβ, which interacts with cell differentiation. [49] 1D11 attenuated an AA-induced increase in interstitial expression of fibronectin around the necrotic tubules (Fig 6D). Fibronectin is secreted early by interstitial fibroblasts, whereas collagen deposition occurs later and is considered as a scarring process. Present findings are in agreement with our previous data reporting progressive increase in collagen type III and I deposition from 10 days of AA intoxication[39]; therefore, we were not surprised to detect a positive effect only on fibronectin immunostaining and not on Picrosirius red staining (collagen deposits).

### Anti-TGFβ decreased cortical expression of PDGF receptor β and VEGF receptor 2

**Fig 7:**
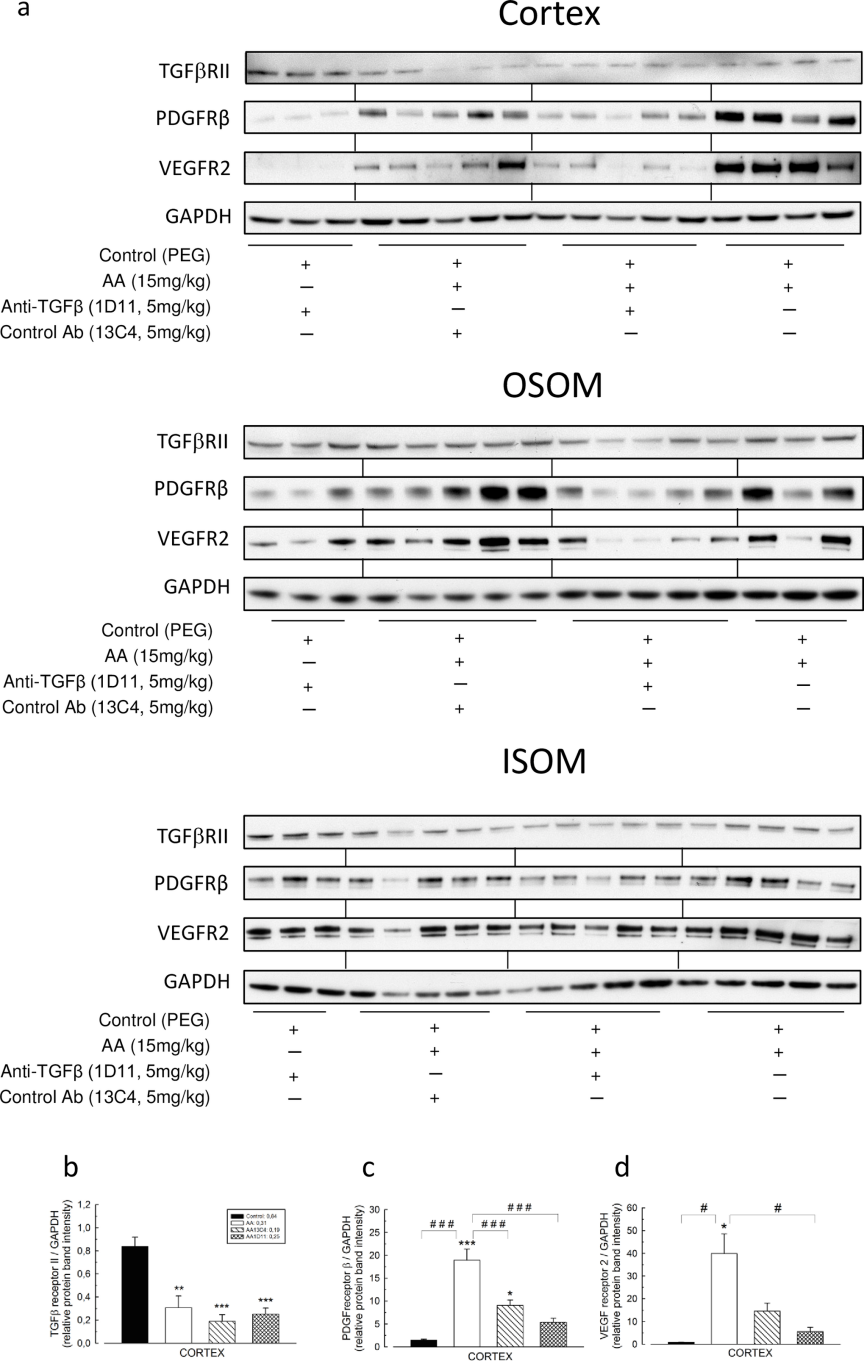
Inhibition of transforming growth factor beta (TGFβ) signaling pathway decreases expression of TGFβ receptor II (TGFRβII), vascular endothelial growth factor receptor 2 (VEGFR2) and platelet-derived growth factor receptor beta (PDGFRβ) induced by aristolochic acid (AA) in kidney cortex. (**a**) Tissue lysates from kidney cortex were immunoblotted for TGFRβII, VEGFR2, PDGFRβ and glyceraldehyd 3-phosphate dehydrogenase (GAPDH) expression. Bands intensities of TGFRβII protein in studied groups (n = 3 for controls; n = 5 for AA and AA+control Ab groups; and n = 4 for and AA+anti-TGFβ group) were quantified by densitometry. (**b**) The control group displayed a basal level TGFRβII expression, and AA intoxication negatively regulated this expression. Bands intensities of VEGFR2 protein in studied groups (n = 3 for controls; n = 5 for AA and AA+control Ab groups; and n = 3 for and AA+anti-TGFβ group) were quantified by densitometry. (**c**) AA highly increases VEGFR2 expression and anti-TGF Ab had a protective effect. Bands intensities of PDGFRβ protein in studied groups (n = 3 for controls; n = 5 for AA, AA+control Ab and AA+anti-TGFβ groups) were quantified by densitometry. (**d**) Anti-TGF Ab and 13C4 had a protective effect on AA induced increase in PDGFRβ protein expression. Results are presented as means ± SEM. One way ANOVA, *** *P* < .001, * *P* < .05 comparison of each group versus control group; followed by Holm Sidak test, ### *P* < .001, # *P* < .05 comparison between all groups.

As both PDGFRβ and VEGFR2 receptors are induced by TGFβ and knowing that fibronectin activates also pericytes, we further investigated the effects of anti-TGFβ on interstitial perivascular cells. To this end, we investigated cortical VEGFR2, PDGFRβ and TGFβ RII protein expression (Fig 7A). As compared to controls, AA significantly suppressed basal expression level of TGFβ RII (0.31 ± 0.19 *vs* 0,84 ± 0.82, *P* < .001) and increased both PDGFRβ and VEGFR2 expression (18.84 ± 2.45 *vs* 1.45 ± 0.23, *P* < .001 and 39.94 ± 8.58 *vs* 0.85 ± 0.12, *P* < .05, respectively) (Fig 7B–7D). As compared to AA group, 1D11 significantly suppressed cortical expression of PDGFRβ (18.84 ± 2.45 *vs* 5.37 ± 0.91, *P* < .001) and of VEGFR2 (39.94 ± 8.58 *vs* 5.60 ± 1.84, *P* < .05) (Fig 7D).

### Anti-TGFβ Ab modulated interstitial accumulation of PDGFRβ+ perivascular cells

**Fig 8:**
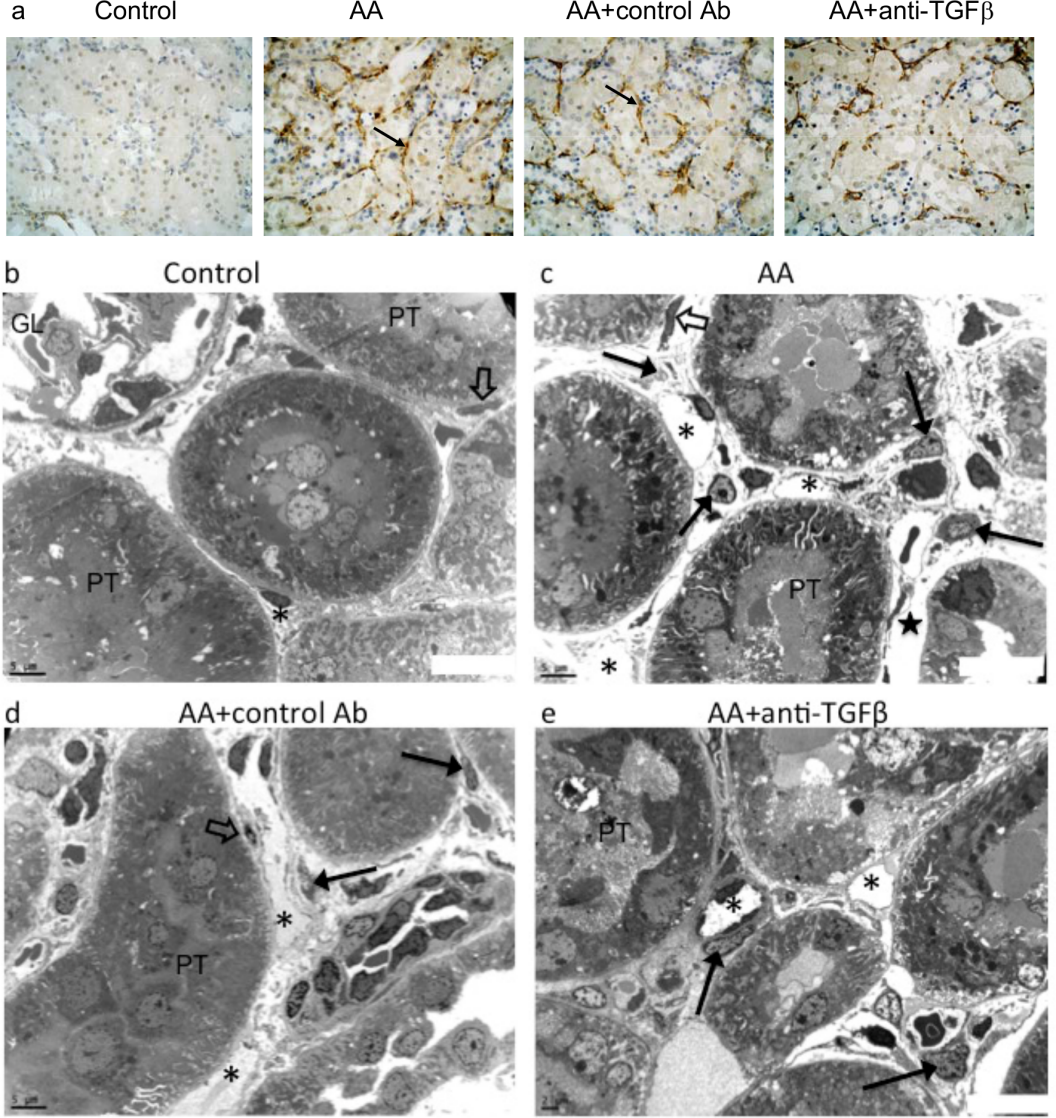
Representative photomicrographs of platelet-derived growth factor receptor beta (PDGFRβ) expression in the kidney tissues. (**a**) Absence of PDGFRβ staining in control rats and enhanced PDGFRβ expression detected around proximal tubules in the areas of medullary rays in kidney section in AA and AA+control Ab, but weak PDGFRβ staining was observed in the anti-TGFβ group. (**b**) In the control group, normal ultrastructure of kidney interstitium, resident fibroblast (open arrow) characterized by elongated nuclei firmly attached to the proximal tubules (PT), peritubular capillary (*). (**c** and **d**) In AA and AA+control Ab groups, increased number enlarged peritubular capillaries (*) with adjacent myofibroblasts (black arrows), obvious interstitial edema (★), (**e**) less severe peritubular capillary injury (*) with closely attached myofibroblasts (black arrows) (F). Original magnifications: (b-d) x2500 and (e) x3000.

As compared to controls, AA treatment increased the number of PDGFRβ+ cells, reflecting pericytes and resident fibroblasts accumulation in the peritubular areas of medullary rays. These cells are closely attached to the peritubular capillaries and TBM. Preconditioning with 1D11 markedly attenuated the expression of PDGFRβ by interstitial cells (Fig 8A). Transmission electron microscopy clearly confirmed the presence of numerous fusiform cells on the abluminal surface of peritubular capillaries in markedly swollen interstitial space (Fig 8B–8D). In fact, anti-TGFβ Ab definitely attenuated peritubular capillary injury, increase in pericytes and peritubular fibroblasts induced by AA (Fig 8A and 8E).

### Anti-TGFβ Ab modulated AA-induced endoplasmic reticulum and mitochondrial stress *in vivo* and *in vitro*

**Fig 9:**
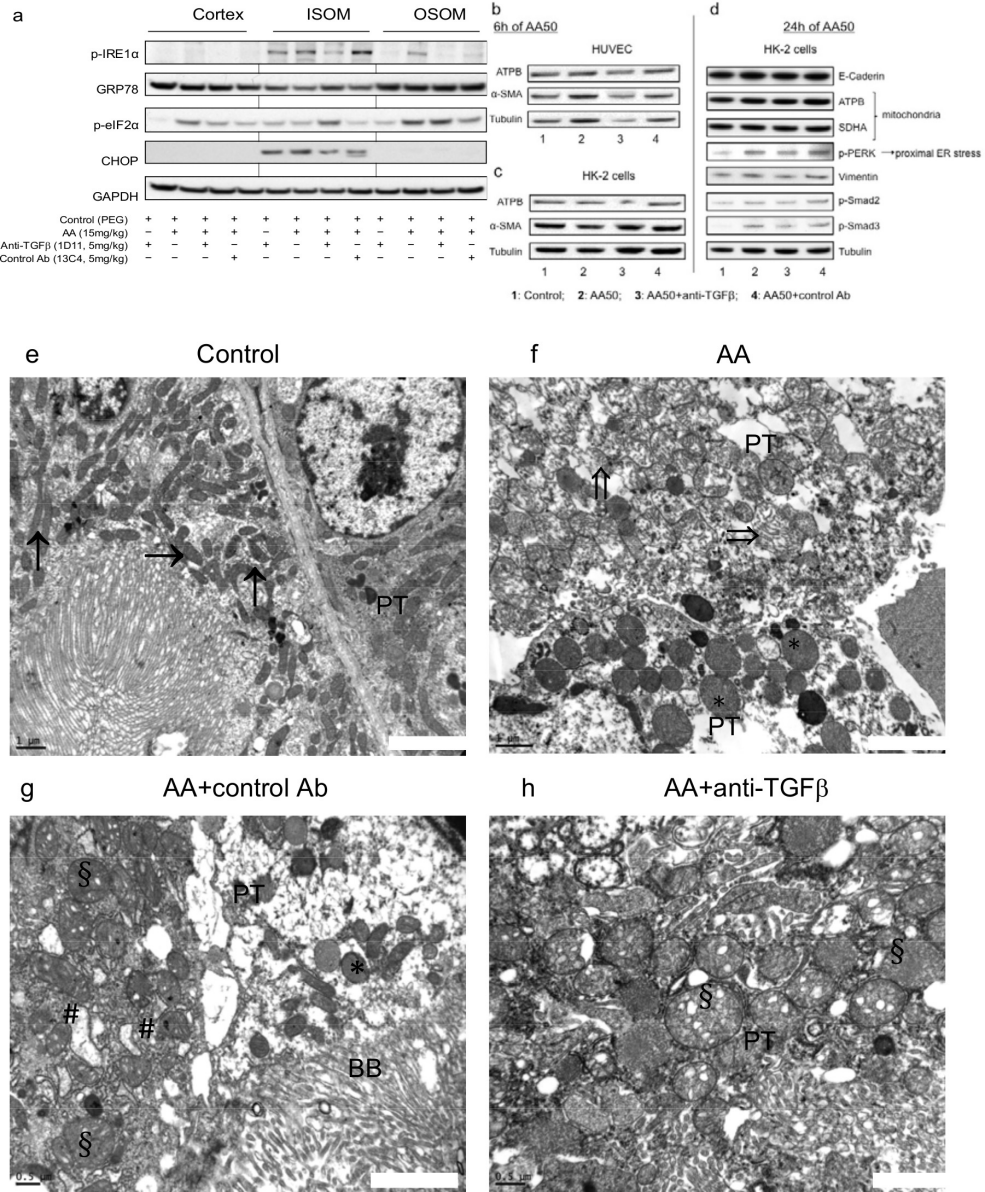
Anti-transforming growth factor beta (TGFβ) Ab decreased aristolochic acid (AA)-induced reticulum endoplasmic (ER) and mitochondrial (MT) stress protein expression in kidney tissue. (**a**) Tissue lysates from kidney cortex, outer stripe of outer medulla (OSOM) and inner stripe of outer medulla (ISOM) were immunoblotted for p-IRE1α, GRP78, p-eIF2α, CHOP and glyceraldehyd 3-phosphate dehydrogenase (GAPDH) expression. Anti-TGFβ Ab decreased AA-induced p-IRE1α expression in the cortex and in the OSOM and down regulated AA-induced p-eIF2α expression in cortex and OSOM; however anti-TGFβ increased p-eIF2α expression in ISOM. Anti-TGFβ markedly attenuated CHOP expression, which was induced by AA only in ISOM. (**b-d**) Cell lysates from HUVEC and HK-2 incubated for 6 and 24 h with 50 ng/ml of AA and anti-TGFβ Ab or control Ab were immunobloted for ATPB, alpha smooth muscle actin (α-SMA), E-cadherin, ATPB, SDHA, p-PERK, vimentin, p-Smad2, p-Smad3, and tubulin proteins expression. (**b-c**) Anti-TGFβ Ab had a protective effect on alpha smooth muscle actin (α-SMA) expression induced by AA after 6 h of intoxication in human umbilical endothelial cells (HUVEC) but not in human proximal tubular cells (HK-2). (**d**) Anti-TGFβ Ab protected proximal tubular cells from AA-induced injury. 1D11 reduced expression of vimentin by decrease of p-PERK reflected proximal endoplasmic reticulum stress induced by AA after 24 h. **Mitochondrial and reticulum endoplasmic (RE) ultrastructure damages attenuated by anti-transforming growth factor β (TGFβ) Ab**. (**e**) Tubular epithelial cells contained normal mitochondria (arrows) in control rats. (**f** and **g**) In AA and AA+control Ab groups, abnormally shaped and focally quite swollen mitochondria (*), disappearance of mitochondria crests and rupture of mitochondria integrity (double arrows) and obvious alterations of RE ultrastructure (swollen RE: #) (**h**) Round, abnormally shaped and swollen mitochondria (§) in proximal tubular cells AA+ anti-TGFβ Ab rat (d). Transmission electron microscopy performed on ultrathin (50 nm) kidney tissue sections, original magnification **e-h** x 20000.

To evaluate the mechanism of mitochondrial and ER stress, tissue lysates from the kidney cortex, the OSOM and the ISOM were immunoblotted for p-IRE1α, GRP78, p-eIF2α, CHOP and GAPDH protein expression (Fig 9A). In the cortex and OSOM, 1D11 decreased AA-induced p-IRE1α expression, a marker of ER stress, and down-regulated AA-induced p-eIF2α expression. Moreover, 1D11 markedly attenuated CHOP expression induced by AA but only in ISOM, reflecting AA-induced mitochondrial injury.

*In vitro* studies confirmed that by p-Smad2/3 signaling pathway inhibition, 1D11 attenuated AA-induced proximal ER stress already after 24 h, as attested by the reduction of p-PERK protein expression in HK-2 cells but not in HUVEC (Fig 9B–9D).

*In vivo*, ultrastructure assessments exhibited severe mitochondria injury and enlarged ER in AA and AA+13C4 groups (Fig 9E–9G). 1D11 markedly reduced not only the extent but also the severity of AA-induced mitochondria and ER injuries (Fig 9H).

### Anti-neoangiogenic effect of anti-TGFβ Ab

**Fig 10:**
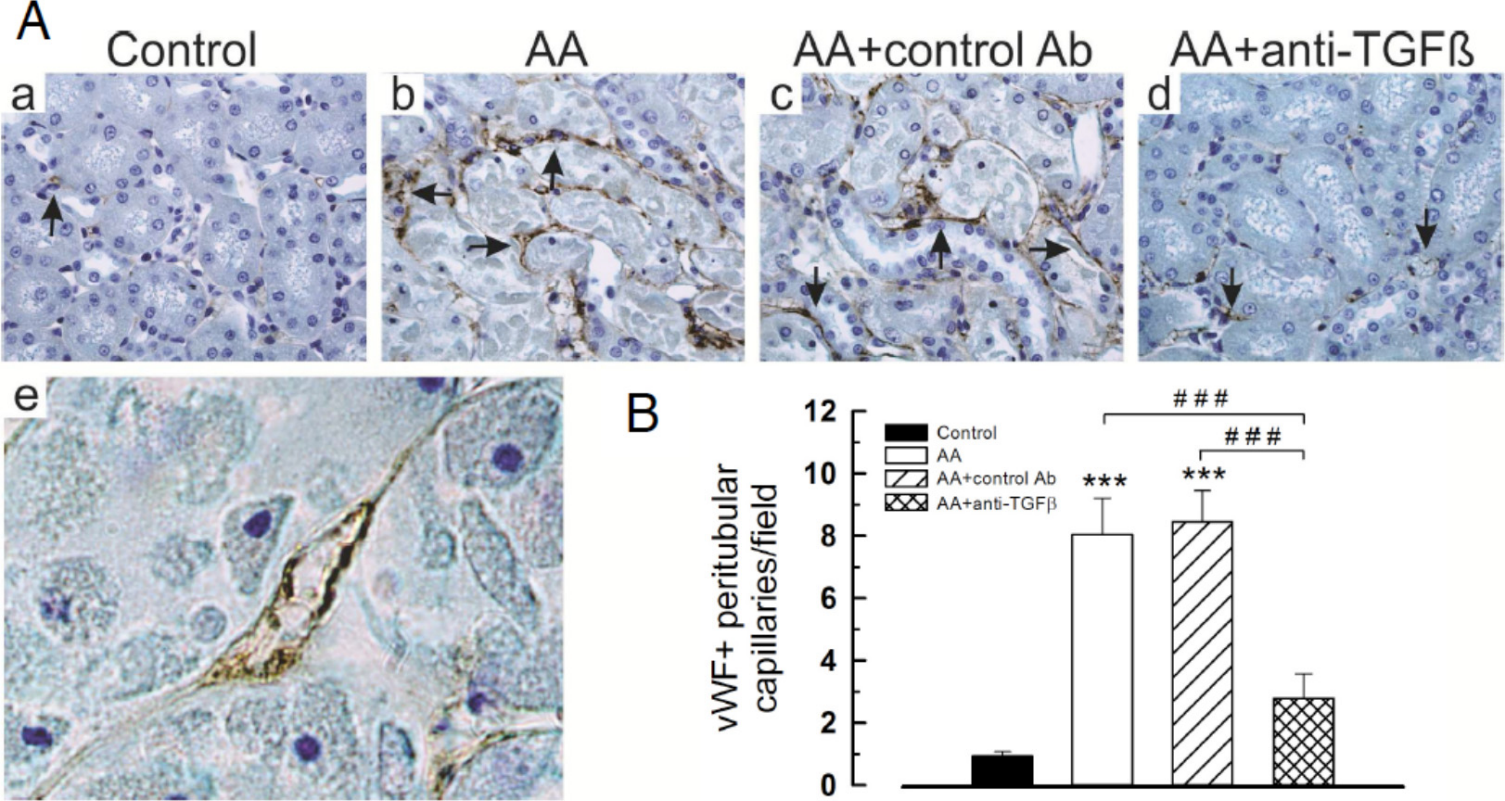
Anti-transforming growth factor beta (TGFβ) Ab attenuated peritubular capillary density *in vivo* and endothelial cells injury *in vitro* induced by aristolochic acid (AA). (**A**) (**a**-**d**) Representative photomicrographs of vWF expression on capillaries (arrows) surrounding injured proximal tubules. Original magnifications **a-d**, x400; **e**: AA+ anti-TGFβ Ab group, x1000. (B) Mean number of vWF+ peritubular capillaries per field. Data are shown as mean ± SEM (n = 6). One way ANOVA, *** *P* < .001 comparison of each group versus controls, followed by Holm Sidak test ^###^ *P* < .001 comparison between groups (n = 6).

As 1D11 modulated cortical expression of VEGFR2, we assessed the density of peritubular capillaries by vWF immunostaining (Fig 10A, a-e and 10B). As compared to controls, AA markedly increased the mean number of vWF-positive peritubular capillaries in the external parts of the medullary rays (8.04 ± 1.17 *vs* 0.94 ± 0.13, *P* < .001). This reflected AA-induced peritubular neoangiogenesis and was significantly reduced by 1D11 (8.04 ± 1.17 *vs* 2.79 ± 0.79, *P* < .001).

## Discussion

The above data question the impact of early TGFβ signaling pathway inhibition as an intervention to prevent PDGFRβ+ perivascular cells activation in AA-induced acute kidney injury in a male Wistar rat model.

Inhibition of p-Smad2/3 signaling pathway has previously been reported to play a critical role in chronic AAN in mice. [50, 51] In our end-stage human AAN several p-Smad2/3, TGFβ receptor or PDGFRβ+ perivascular cells predominated in the zones neighboring, but not within areas of firm fibrosis. Consequently, chronic AAN appeared to us less adequate for our purposes to study initial events of renal fibrosis. In the present model increased urinary excretion of the active form of TGFβ and expression of p-Smad2/3 have been reported after 3 days of AA exposure. Activated epithelial and/or endothelial cells early trigger transition of adjacent perivascular cells into myofibroblasts through paracrine secretion of TGFβ. [38, 39] Therefore, we focused on the acute phase of AAN in rats. The activation of the epithelial-endothelial axis as the initial event in AKI is of particular therapeutic importance because it provides the attractive possibility of a time-dependent selective modulation of perivascular cells activation. [52, 53] In this setting, our above data are of interest as preconditioning of rats with 1D11 attenuated AA-induced functional and structural AKI onset (PCr increase, PTEC necrosis, peritubular capillaritis, interstitial inflammation, PDGFRβ+ perivascular cells accumulation and mitochondrial and RE stress in tubular epithelial cells).

Until now, direct PTEC tubulotoxicity has been considered as the primary insult of AA. [38, 41, 48] However, regarding typography of acute TI lesions limited to the medullary rays, ^34^^,^ ^48^ renal peritubular microvasculature injury seems to be also involved in AAN as it has been previously evoked. [54] Decreased expression of VEGF has been suggested as a mechanism of AA-induced fibrosis in rats [55] and agreed with rarefaction of peritubular capillary network reported in human end-stage AAN. [37] These results contrast with the proangiogenic profile (increased synthesis of VEGF) described in porcine kidney epithelial cells incubated with non-toxic doses of AA. [56] In this context, we reported here two intriguing observations. Firstly, in our human case of AAN, the epithelial cells from atrophic tubules still expressed VEGF. Secondly, acute AA intoxication of rats led to an increase in peritubular capillary density corresponding to neoangiogenesis, which was inhibited by 1D11. These vascular events reflect a mandatory step of wound healing and could be regarded as an attempt of TGFβ-mediated repair actions. [22, 57, 58] Nevertheless, increased delivery of oxygen to injured tubular epithelial cells [45, 48] could increase AA-induced cortical oxidative stress. [38] Consequently, neoangiogenesis appears to be harmful in our model of AKI as 1D11 significantly reduced neoangiogenesis and improved renal function. In fact, for unknown reasons, very early and probably coinciding with trespassing over the point of no return, initial repair actions become distorted by the pathological scenario and turn TGFβ into a key factor of CKD progression. [59]

Our present findings underline that beside PTEC, epithelial cells from the loop of Henle and EC from peritubular microvasculature appear as new cellular targets of AA and as underestimated players in AAN. Both ATN and loss of peritubular capillary sealing by endothelium injury are plausible mechanisms explaining severe interstitial edema and polyuria, which did not occur in rats treated by 1D11. Descending arterial *vasa recta* in OM have a non-fenestrated endothelium with a *zona occludens* limiting permeability. They express water channels and facilitate urea transport involved in the concentration of urine.[60] PTEC intoxicated by AA are a main source of TGFβ [38, 56] leading to activation of adjacent fibroblasts and endothelial cells. [42, 56] Injured epithelial cells from TAL activate pericytes throughout adenosine secretion. [61] On the other hand, independently of local inflammation, AA primarily incites TGFβ release from injured EC that further alter tubular epithelial cells and activate pericytes [62] leading to peritubular fibrosis by generation of myofibroblasts. [57, 63] The immunostaining of αSMA actually labeled different components such as myofibroblasts and also vessel walls, whereas Pircosirus red staining labeled the collagen fibers. Quantification of αSMA expression was performed at a small magnification, allowing us to screen large areas of kidney parenchyma. In contrast, the evaluation of Picrosirius red staining was mainly done in the external part of the medullary rays where most of the tubulointerstitial changes are seen in AAN (interstitial fibrosis and tubular atrophy reported in chronic phase of AAN). These differences in specificity of labeling and methodology of quantification could explain the discrepancy between αSMA expression and extracellular matrix deposition.

Anti-TGFβ antibody prevented AA-induced PDGFRβ+ perivascular cell accumulation accordingly to the hypothesis that TGFβ mediates the progression of renal diseases through destabilization of the microvascular network. [64] In hypertensive Dahl salt sensitive rats, 1D11 has also been reported to protect the integrity of the vascular bundles. [28] Endothelial cells in these vessels are more vulnerable to injury than those from peritubular capillaries as they are exposed to an osmolality of up to 800 mOsm and low oxygen content. [16] In efferent arterioles forming a peritubular capillary network, a discontinuous layer of pericytes is known to progressively replace the outer smooth muscle layer [60] resulting in higher pericytes density in OM than in inner medulla. [61] In this setting, our data of p-Smad 2/3 signaling affected by 1D11 predominantly in ISOM, imply that EC from vascular bundles and/or epithelial cells from the loop of Henle are probably additional AA targets. Direct endothelial cells injury by AA, capillary pericytes dissociation form their blood vessels and their transition into the myofibroblasts are well-recognized factors reducing endothelial cells survival and capillary density observed also in end-stage AAN. [37, 57]

Defective UPR pathways, which highly alter the function and viability of renal cells, are involved in the progression of various kidney diseases. [6] Our *in vivo* and *in vitro* data showed severe mitochondrial and ER ultrastructure damage, which was correlated with dysregulation of UPR pathways. All of these events were attenuated by 1D11, suggesting these beneficial effects are related, at least partly, to the maintenance of ER and mitochondrial proteostasis.

It has been recognized that ISOM has higher capacity for anaerobic metabolism, which may make it more susceptible to oxidative injury but also implies that compensatory responses to injury may differ from the OSOM response. Whereas phosphorylation of eIF2a is generally thought to translate as a reduction in global protein synthesis, recent data suggest selective regulation of translation during stress that is independent of the 5' cap structure. [65] We suggest that the field is sufficiently unsettled to be able to interpret this result and given the very different metabolic profile of the ISOM and OSOM, we are careful to interpret the meaning of eIF2 phosphorylation, as selective translation may be part of the compensatory response.

**Fig 11:**
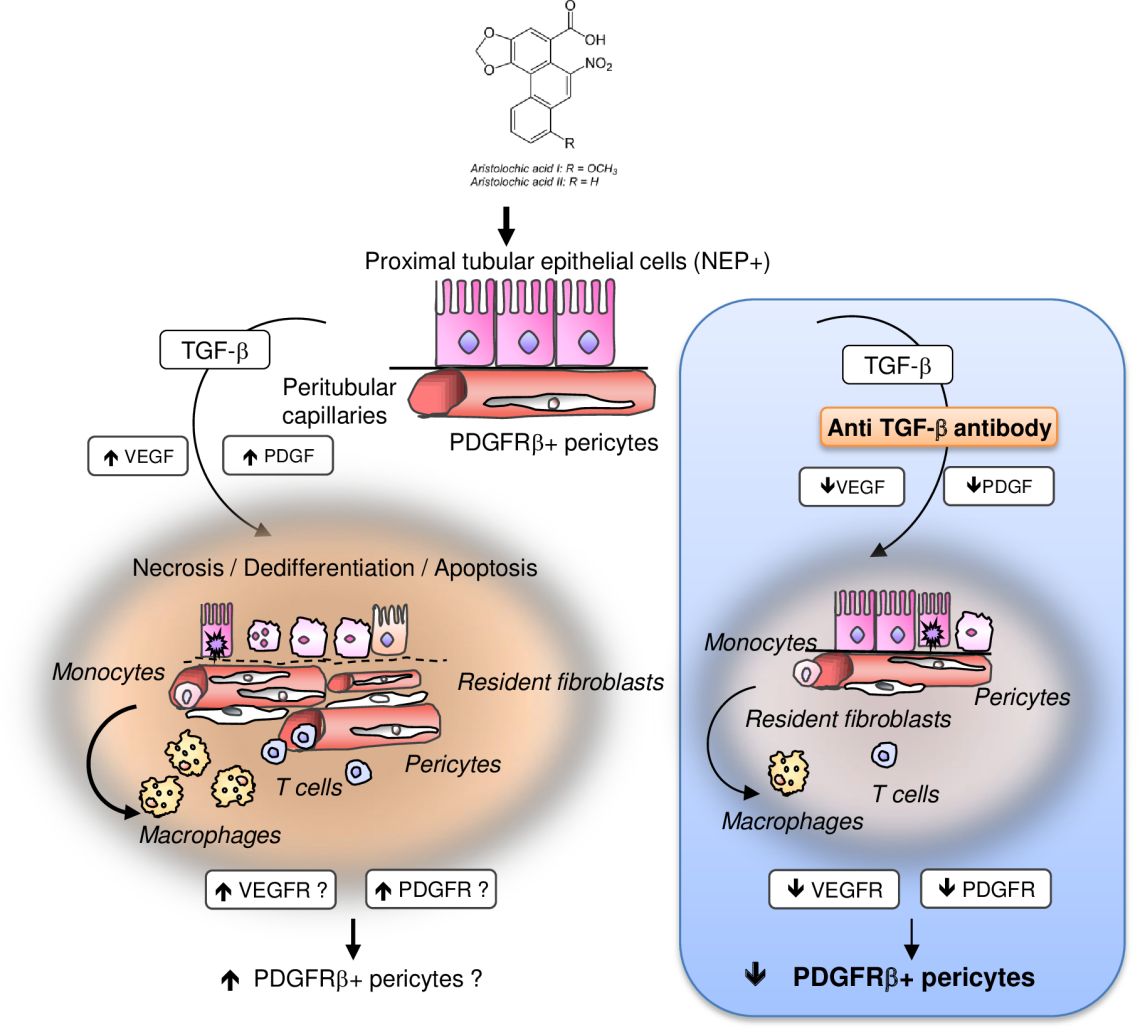
Proposed mechanism of transforming growth factor beta (TGFβ) involvement in early activation of PDGFRβ+ pericytes in aristolochic acid induced acute kidney injury in Wistar male rats.

We recognize that our results reflect only a static snapshot of a very dynamic fibrotic process. In contrast to unaffected expression TGFRII protein (resident fibroblasts), 1D11 significantly reduced VEGFR2 and PDGFRβ protein expression. Therefore, we propose that PDGFRβ positive pericytes and not resident fibroblasts are the main source of myofibroblasts in AA-induced AKI (Fig 11). This could explain that selective abrogation of TGFRII signaling pathway is not sufficient to reduce renal fibrosis 5 weeks after AA-induced AKI in mice. [66] We agree that the role of TGFβ inhibition in driving fibroblast persistence *“in vivo”* in the rodent kidney remains a crucial question. Investigating the chronic phase of experimental AAN would give some insights about the impact of early preconditioning with TGFβ on interstitial fibrosis, the late onset of AA toxicity. However, our aim in the present study was to assess the impact of TGFβ inhibition on the outcome of renal function in early steps of AA exposure, focusing on very early ultrastructure alterations of epithelial-endothelial axis and interstitial perivascular cells activation. For these reasons, we decided to investigate the acute phase of AAN in our rat model. The effect of TGFβ inhibition still remains controversial also in this context.

## Conclusions

The early inhibition of p-Smad2/3 signaling pathway improved impairment of renal function in AA-induced acute kidney injury in rats. In our hand, preconditioning with neutralizing anti-TGFβ antibody (1D11) partially prevented epithelial-endothelial axis activation by maintaining cellular proteostasis and reduction of pericytes-derived PDGFRβ+ perivascular cells accumulation. This is an evolving field and the interpretation of this finding requires further study.

## Abbreviations

AA: Aristolochic acid
AAN: Aristolochic acid nephropathy
Ab: Antibody
AKI: Acute kidney injury
CKD: Chronic kidney disease
EC: endothelial cells
EMT: epithelial-to-mesenchymal transition
EndoMT: endothelial-to-mesenchymal transition
ER: endoplasmic reticulum
HUVEC: human umbilical vein endothelial cell
ISOM: Inner stripe of outer medulla
NEP: Neutral endopeptidase
OSOM: Outer stripe of outer medulla
PDGFRβ: Platelet-derived growth factor receptor-beta
PCr: Plasma creatinine
PTEC: Proximal tubular epithelial cell
SMA: smooth muscle actin
TBM: tubular basement membrane
TGFβ: Transforming growth factor-beta
TI: Tubulointerstitial
UPR: Unfolded protein response
VEGF: Vascular endothelial growth factor
vWF: Von Willebrand Factor
